# Preventive actions of allergen immunotherapy: the facts and the effects in search of evidence

**DOI:** 10.1186/s12948-017-0070-7

**Published:** 2017-06-15

**Authors:** Irene Martignago, Cristoforo Incorvaia, Erminia Ridolo

**Affiliations:** 1 Medicine and Surgery Department, University of Parma, via Gramsci n.14, 43126 Parma, Italy; 2 ASST Pini/CTO, Cardiac/Pulmonary Rehabilitation, Milan, Italy

**Keywords:** Allergen immunotherapy, Preventive capacity, Mechanisms of action

## Abstract

Allergen immunotherapy (AIT) is the only treatment that works on the causes of allergy. Available AIT nowadays are subcutaneous immunotherapy (SCIT) and sublingual immunotherapy (SLIT) for allergic rhinitis and asthma, while for allergy to *Hymenoptera* venom only subcutaneous route is recommended. A bulk of trials and meta-analyses demonstrated that efficacy and safety of AIT in decreasing allergic clinical symptoms and use of rescue medications, while its preventive capacity is yet under investigation. The most important of these effects is the prevention of potentially fatal anaphylactic reactions to *Hymenoptera* stings by venom immunotherapy (VIT). A certain number of studies thus far available showed that AIT, in both forms, is able to prevent the progress of allergic rhinitis into asthma and the development of new sensitizations. These effects should be related to the mechanisms of action of AIT. In fact, it has been demonstrated that both SCIT and SLIT are able to modify the allergen presentation by dendritic cells, with result in modification of the phenotype of allergen-specific T cells, switching from the typical of allergic inflammation Th2-type response to a Th1-type one. Also allergen-specific T regulatory (Treg) cells play a pivotal role by producing suppressive cytokines, such as IL-10 and TGF-beta. However, the only plain evidence of a preventive effect concerns VIT, while the other outcomes need to be furtherly investigated.

## Background

Allergen immunotherapy (AIT) dates back to 1911 when Leonard Noon ideated a new therapy for hay fever, that consisted of administering increasing doses of a raw extract of grass pollen [1]. Noon speculated that pollens contained toxins and their injection to patients would have to make them “immunized”, like a vaccine, as it was started to know about infectious diseases. Obviously, aetiology and pathophysiology of allergy was then largely unknown. The very first clinical trial was performed only in 1954 by Frankland [2, 3]. The term “vaccine” recurred again in a World Health Organization document about AIT in 1998: the reason to suggest it was “to reflect the fact that allergen vaccines are used in medicine as immune modifiers similarly to vaccines for infectious diseases” [4]. However, this therapy is currently commonly known as AIT.

Subcutaneous immunotherapy (SCIT) was the only way of AIT administration until the 1980s, but a case-series of deaths after this treatment, occurred both in Europe [5] and in the USA [6], raised the important issue of treatment safety. Actually, recent data estimated that one life-threating reaction occurred in US every 2.5 million injections, which is equivalent to 3.4 deaths per year [7]. Moreover, if the contraindications in prescribing SCIT and the adequate protocols of administration are respected, the rate of systemic reactions is around 0.1–0.2% [8]. Anyhow, sublingual immunotherapy (SLIT) is now a consolidated and safe practice, with proved efficacy as demonstrated by several trials [9–11]. Besides, SLIT presents a very good profile of safety with only an average of 11 anaphylaxis in 1 billion of SLIT doses since 2000 [12].

Nowadays, SCIT and SLIT are available for allergic rhinitis (AR) and allergic asthma (AA) due to sensitization to aeroallergens such as pollens and house dust mites (HDM), while SCIT is the only recommended route of administration for *Hymenoptera* venom allergy. Since a bulk of literature has been produced proving the efficacy and safety of SCIT and SLIT, the aim of this review is to focus the preventive capacity of AIT.

## Prevention of allergic reactions to insect stings

About 3% of the general adult population presents systemic reactions to stings of insects belonging to the order of *Hymenoptera*, including honeybees (*Apis spp*), bumblebees (*Bombus spp*), yellow jackets (*Vespula spp*), wasps (*Polistes spp*), and hornets (*Vespa spp and Dolichovespula spp*). Systemic reactions after *Hymenoptera* stings in allergic patients can cause anaphylaxis, with a risk of potentially fatal outcome [13]. SCIT with *Hymenoptera* venom, known as venom immunotherapy (VIT) is highly effective in preventing further systemic reactions, as reported in a systematic review [14]. VIT is also very safe, since no fatalities after VIT have been reported. Moreover, most of the adverse reactions are usually mild and the necessity of administering adrenalin is rare [15]. Also the rate of systemic reactions to stings in treated patients is globally low, corresponding to around 3% in adults [16]. For reasons yet to be completely understood, honeybee VIT has an higher incidence of systemic reactions, that is fivefold more frequent than for wasp venom, especially in build-up phase of VIT [17]. Korosec et al. [18] identified as risk factor for severe systemic reactions a short latency from sting and low levels of sIgE for rApi m1. Besides an high profile of safety, VIT is also associated with prevention of fatal reaction in 100% of patients and of any kind of systemic reactions in 90–95% [19]. In case complete protection is not achieved, increasing the maintenance dose of venom over the standard of 100 μg is recommended and usually permits to reach full protection [20]. Failure to reach protection is more common in VIT with bee venom than in the one for vespid. Recently Frick et al. [21] identified sensitization to Api m10 as a risk factor for failure of VIT. Author identified in patients nonresponder to VIT high levels of IgE for Api m10. Correspondingly, low levels of Api m10 were detected in the extracts used for VIT.

Of note, recent data proved that VIT is safe and efficacy also in patients suffering from mastocytosis [22, 23], making VIT an irreplaceable therapy in this condition.

In conclusion, efficacy and safety of VIT are well established, improving also the disease-specific quality of life of these patients, as reported in a recent review [15].

## Prevention of asthma in patients with allergic rhinitis

AR is often associated with allergic asthma, according to the concept of united airway disease [24], and up to 50% of patients with AR can present bronchial hyper-reactivity [25].

The first study to evaluate the preventive effect of SCIT in the development of asthma was conducted in 2000 by Grembiale et al. [26] who studied the effect of SCIT for HDM in a small group of 22 patients with AR and bronchial hyper-reactivity demonstrating that after 2 years of treatment none of them presented asthma.

A large prospective randomized controlled study, the preventive allergy treatment (PAT) study [27], confirmed these results, with a preventive effect persistent over the 10-years period of follow up. The PAT study evaluated children aged between 6 and 14 years, with grass and/or birch pollen allergy and treated for 3 year with SCIT; 24 of 53 control patients developed asthma against 16 of 64 SCIT-treated children. The longitudinal treatment effect, adjusted for bronchial hyper-responsiveness and asthma status at baseline, was statistically significant (P = 0.0075). The odds ratio for no-asthma was 4.6 (95% CI 1.5–13.7) in favour of AIT [28].

Regarding SLIT, the preventive effect on asthma has been also investigated in a prospective study on 60 children allergic to HDM [29]. Patients were divided in two groups: 35 treated with SLIT for 4–5 years and 25 treated only with drugs. Subjects in the treatment group presented a reduced incidence of asthma (P < 0.01), while no difference was observed in the control group. These results were confirmed in a study by Marogna et al. [30]: mild persistence asthma was less common in patient treated with SLIT respect than patients receiving only symptomatic drugs (OR 0.04%); besides, children with a positive methacholine challenge were reduced in number after 3 years of SLIT.

A recent observational open study performed in real life by Marogna et al. [31] confirmed these results in 75 patients (mean age 27 years) with AR to HDM and treated with SLIT, assessing a preventive effect on pulmonary function over a 5-year period (P < 0.001). In fact, the authors evaluated not only the development of asthma form the clinical point of view, but also by monitoring FEV1 values and reactivity to methacholine challenge test (P < 0.001).

Also the preventive effect of SLIT for pollen has been evaluated. An open randomized study on the effect of grass-pollen SLIT evaluated 47 children, who were treated for 3 years. Compared to them, the control group, that underwent only symptomatic drug therapy, presented a 3.8 times greater risk of developing asthma [32]. AIT with tablets and its role in asthma prevention was investigated in the GAP trial [33] a double-blind placebo-controlled trial including 812 children with grass pollen-induced rhinoconjunctivitis. After 5 years of therapy with SQ-standardized grass pollen tablet, children in the treatment group presented a reduced risk of developing asthma compared to placebo group (Odds Ratio 0.71, P < 0.05) [34]. The available studies are listed in Table 1.

**Table 1:** Prevention of asthma

Study	No patients	Allergen	AIT	AIT—years	Follow-up
Grembiale et al. [26]	22 Adults	House dust mites	SCIT	2	NA
Niggemann et al. [27] Jacobsen et al. [28]	117 Children	Grass pollen Birch pollen	SCIT	3	10 years
Di Rienzo et al. [29]	60 Children	House dust mites	SLIT	4–5	10 years
Marogna et al. [30]	216 Children	NA	SLIT	3	NA
Marogna et al. [31]	124 Children and adults	House dust mites	SCIT and SLIT	5	NA
Novembre et al. [32]	113 Children	Grass pollen	SLIT	3	NA
Valovirta et al. [33]	812 Children	Grass pollen	SLIT tablet	5	NA

*AIT* allergen immunotherapy, *SCIT* subcutaneous immunotherapy, *SLIT* sublingual immunotherapy, *NA* Not available

According to a recent systematic review, both SCIT and SLIT have an evidence of a significant preventive effect in reducing the development of asthma [35].

## Prevention of new sensitizations

Developing new sensitizations is very frequent in the natural history of respiratory allergy. A preventive effect of AIT on the onset of new sensitizations is reported in position papers and consensus documents, based on the findings from some studies [30, 36, 37].

However, data available yet are far from clear about this effect. In fact, some recent reviews reported a globally low grade of evidence. Kristiansen et al. [35] indicated that there is some evidence in favor of a reduced risk of developing new sensitizations at least over the short period, but none on the long-term. Di Bona et al. [38] stated that preventive effect has not been proved yet, but that high-quality studies could refute this result. In fact, evidences of this preventive effect are derived from retrospective or nonrandomized studies [39].

In addition, often such studies included too small groups of patients to provide statistical robustness to the findings [40–43].

## Is conceivable a primary prevention of respiratory allergy by AIT?

The role of AIT in prevention of allergy in children with an high risk of developing food or aeroallergen sensitizations was recently investigated. The first study was conducted by Holt in 50 children aged between 18 and 30 months and at least on food allergen sensitization. Children were treated daily for 1 year with a mixture of HDM, cat and timothy grass allergens, to be taken orally. Results obtained thus far in the follow-up did not demonstrate a significant differences in sensitizations or asthma between AIT and control groups [44]. A similar approach was tried by Zolkipli et al. [45] in 111 young children (aged 5–9 months), with negative skin prick tests but an high risk of atopy, who were treated with dust mites SLIT. There was a significant (P = 0.03) reduction in sensitization to any common allergen in the active (9.4%) compared with placebo (25.5%) treatment groups. The results met the trial’s pre-specified criteria for proof of concept in reducing sensitization to any allergen, but, no significant preventive effect was observed on HDM sensitization or allergy-related symptoms. A recent randomized double-blind placebo-controlled trial was also conducted in a small group of adults [46]. The authors demonstrated that SLIT for cedar pollen administered to sensitized but asymptomatic subjects was effective in preventing the development of allergic symptoms.

## The mechanisms underlying the preventive capacity of AIT

The immunological mechanisms underlying the response to AIT are very complex and not yet completely elucidated. They can be synthesized in 3 phases [47]:

1. rapid desensitization, characterized by a fall in degranulation of mast cell and basophils, probably secondary to un upregulation of histamine type 2 receptor, even in presence of augmented allergen-specific IgE levels [48];
2. early tolerance, defined by decrease of IL-4 secreting Th2 cell and increase of IL-10 secreting inducible Treg cells, together with the increase of Breg cell [49]. In fact, the allergen, administered by subcutaneous or oral route, is captured by dendritic cells that influence a modification of the phenotype of allergen-specific T cells, with a switch from the typical of allergic Th2-type response to a Th1-type one. As a result, there is an increased production of IL-10 and TGF- beta [47] and an augmented level of inducible Treg cells, that is correlated to the improvement of allergic symptomatology [50];
3. sustained tolerance, that is mediated by changes in memory T- and B-cells. Treg cells stimulate B cells to the class-switching towards IgG, tolerogenic allergen-specific antibodies that compete with IgE for allergen binding, avoiding the contact between the specific allergen and the IgE on the surface of mast cells and basophils. Thus, mediators are not released by these cells when in contact with the allergen [51].

Recent data focus on the possibility of a generation of IgE blocking the activity of IgG, leading to a negative AIT outcome [52, 53].

## Conclusions

AIT for respiratory allergies has a huge amount of evidence as regards efficacy and safety, in both forms of SCIT and SLIT, as supported by meta-analyses [11] and, for the latter, by the recent trials including very large populations of patients that make meta-analysis unwarranted [54]. As far as the preventive role of AIT is concerned, the only plain evidence can be recognized to VIT in the prevention of fatal reactions to *Hymenoptera* stings. As to the other preventing actions, there is some evidence for SCIT in decreasing the risk of developing asthma in children with AR, while the possible prevention of new sensitizations by both SCIT and SLIT requires a higher level of evidence. Regarding the fascinating possibility of AIT as a prophylactic instrument in children at high risk of developing atopy and in patients sensitized but not yet symptomatic, more studies are needed to envision such a role that, if confirmed, could influence the epidemiology of allergy.

## Abbreviations

AIT: allergen immunotherapy
SCIT: subcutaneous immunotherapy
SLIT: sublingual immunotherapy
VIT: venom immunotherapy
Treg: T regulatory
AR: allergic rhinitis
HDM: house dust mites
